# Towards Therapeutic Alternatives for Mercury Neurotoxicity in the Amazon: Unraveling the Pre-Clinical Effects of the Superfruit Açaí (*Euterpe oleracea*, Mart.) as Juice for Human Consumption

**DOI:** 10.3390/nu11112585

**Published:** 2019-10-26

**Authors:** Maria Elena Crespo-López, Ericks Sousa Soares, Barbarella de Matos Macchi, Leticia Santos-Sacramento, Priscila Yuki Takeda, Amanda Lopes-Araújo, Ricardo Sousa de Oliveira Paraense, José Rogério Souza-Monteiro, Marcus Augusto-Oliveira, Diandra Araújo Luz, Cristiane do Socorro Ferraz Maia, Hervé Rogez, Marcelo de Oliveira Lima, João Paulo Pereira, Diomar Cavalcante Oliveira, Rommel Rodrigues Burbano, Rafael Rodrigues Lima, José Luiz Martins do Nascimento, Gabriela de Paula Arrifano

**Affiliations:** 1 Laboratory of Molecular Pharmacology, Federal University of Pará, Belém-PA 66075-110, Brazil; ericks.sousa@gmail.com (E.S.S.); yukitakeda98@gmail.com (P.Y.T.); amanda.lopes1647@gmail.com (A.L.-A.); pos_ricardo@hotmail.com (R.S.d.O.P.); rogerio.souza.monteiro@gmail.com (J.R.S.-M.); marcusoliveira@globo.com (M.A.-O.); gabrielaarrifano@uol.com.br (G.d.P.A.); 2 Laboratory of Neurochemical Investigation, Center of Biological Sciences, Federal University of Santa, Catarina, Florianópolis–SC 88040-900, Brazil; 3 Laboratory of Molecular and Cellular Neurochemistry, Federal University of Pará, Belém-PA 66075-110, Brazil; bmacchi@gmail.com (B.d.M.M.); jlmn@ufpa.br (J.L.M.d.N.); 4 Laboratory of Experimental Neuropathology, Department of Pharmacology, University of Oxford, Oxford OX1 3QT, UK; 5 Laboratory of Pharmacology of Inflammation and Behavior and Federal University of Pará, Belém-PA 66075-110, Brazil; diandra.arluz@gmail.com (D.A.L.); crismaia@ufpa.br (C.d.S.F.M.); 6 Centre for Valorisation of Amazonian Bioactive Compounds (CVACBA) and Federal University of Pará, Belém-PA 66075-110, Brazil; herverogez@gmail.com; 7 Evandro Chagas Institute, Secretary of Sanitary Surveillance, Belém-PA 66093-020, Brazil; marcelolima@iec.pa.gov.br (M.d.O.L.); joaopereira@iec.pa.gov.br (J.P.P.); diomarcavalcante@iec.pa.gov.br (D.C.O.); 8 Laboratory of Molecular Biology, Ophir Loyola Hospital, Belém-PA 66063-240, Brazil; rommel@ufpa.br; 9 Laboratory of Functional and Structural Biology, Federal University of Pará, Belém-PA 66075-110, Brazil; rafalima@ufpa.br

**Keywords:** açaí, acai, aging, antioxidant, Euterpe, extract, intoxication, methylmercury, telomere

## Abstract

Methylmercury (MeHg) exposure is a serious problem of public health, especially in the Amazon. Exposure in riverine populations is responsible for neurobehavioral abnormalities. It was hypothesized that consumption of Amazonian fruits could protect by reducing mercury accumulation. This work analyzed the effects of commercial samples of *Euterpe oleracea* (EO) for human consumption (10 μL/g) against MeHg i.p. exposure (2.5 mg/Kg), using neurobehavioral (open field, rotarod and pole tests), biochemical (lipid peroxidation and nitrite levels), aging-related (telomerase reverse transcriptase (TERT) mRNA expression) and toxicokinetic (MeHg content) parameters in mice. Both the pole and rotarod tests were the most sensitive tests accompanied by increased lipid peroxidation and nitrite levels in brains. MeHg reduced TERT mRNA about 50% demonstrating a strong pro-aging effect. The EO intake, similar to that of human populations, prevented all alterations, without changing the mercury content, but avoiding neurotoxicity and premature aging of the Central Nervous System (CNS). Contrary to the hypothesis found in the literature on the possible chelating properties of Amazonian fruits consumption, the effect of EO would be essentially pharmacodynamics, and possible mechanisms are discussed. Our data already support the regular consumption of EO as an excellent option for exposed Amazonian populations to have additional protection against MeHg intoxication.

## 1. Introduction

Mercury is a hazardous metal, very useful for industry applications because it is a liquid (at room temperature) capable of conducting electricity. The organic form, methylmercury (MeHg), is the most toxic mercury compound responsible for episodes of human intoxication [1]. Exposure to MeHg is a serious public health problem throughout the world and especially in the Amazon [1,2]. One of the main anthropogenic sources of mercury in the Amazon is the artisanal and small-scale gold mining (ASGM), which usually uses mercury to extract the gold particles from the river sediments. Then, the mixture of mercury and gold is heated to separate both metals, with the consequent evaporation of part of the mercury that contaminates the surrounded environment. According to the Amazon Geo-referenced Socio-environmental Information Network (RAISG, in Portuguese), there are presently more than 2300 mining sites in the Amazon (available at: https://www.amazoniasocioambiental.org/en/). Additionally, Amazonian soils may be naturally very rich in this metal [2,3,4] and anthropogenic alterations of the environment, such as river damming, may provide adequate conditions for bacteria proliferation and the metal extraction from the soils. Mercury from anthropogenic and natural sources can be transformed into MeHg by methanogenic bacteria and then bioaccumulated through food chain. Thus, human exposure to mercury in the Amazon is detected even in areas where no ASGM is present [5].

Human populations are exposed to MeHg mainly by the consumption of contaminated fish [1,2,6]. In the Amazon, for example, contaminated fish for human consumption was already detected in both urban and riverine areas [2,6]. Amazonian populations are chronically exposed to this metal, presenting current levels as high as 75 µg/g of hair mercury [2,5,7,8]. To understand this exposure, the daily oral intake of MeHg recommended by the WHO and US EPA may be considered as approximately equivalent to 1.8 and 1.2 µg/g of hair mercury respectively, according to the conversion factors previously developed [9,10,11]. The human exposure worldwide is such a matter of concern that an international treated entered into force on 16 August 2017, the Minamata Convention on Mercury (www.mercuryconvention.org), with the endorsement of 129 countries (including Brazil), along with the World Health Organization (WHO). This action supports the worldwide effort to reduce and combat environmental and human exposure to this metal.

The main target organ of MeHg is the central nervous system (CNS). Despite the possible differences of mercury accumulation between the CNS areas, this metal is widely distributed in the brain [12,13]. MeHg neurotoxicity is characterized mainly by motor disorders such as paresthesia, alterations of motor coordination and visual dysfunction [1]. Exposure to mercury levels, such as those in the Amazonian riverine populations, is able of causing behavioral abnormalities even with low doses [14,15,16]. For example, Mediterranean populations with median hair mercury of 9.6 µg/g and consumers of tuna fish, demonstrated significantly worse neurobehavioral performance on color word reaction time, digit symbol reaction time and finger tapping speed [14]. Additionally, the presence of tremors was already associated to mercury exposure in populations with a fish-based diet [15]. In Amazonian populations, high rates of several symptoms such as paresthesia, muscular weakness and tremors were recently showed [16].

The MeHg neurotoxicity is closely linked to oxidative stress in the brain. This metal increases the generation of free radicals (oxygen and nitrogen reactive species) responsible for processes such as lipid peroxidation and genotoxicity [1,17,18]. Therefore, antioxidants compounds and extracts are usually tested as therapies against MeHg neurotoxicity. Moreover, it was hypothesized that the consumption of Amazonian fruits would protect exposed populations, reducing the accumulation of mercury in humans [19]. Despite of that, studies with Amazonian fruits and MeHg intoxication are relatively scarce, being mainly limited to fruits such as guaraná, maná-cubiu, burití and açaí [20,21,22,23,24]. Although guaraná (*Paullinia cupana*, Mart.) already demonstrated that promotes the expression of antioxidant genes in *C. elegans* [20], locomotor improvement in fruit flies [21] and in vitro partial protection against neuroinflammation caused by MeHg [21], no data in mammals is still available. However, maná-cubiu (*Solanum sessiliflorum* Dunal), burití (*Mauritia flexuosa* L.) and açaí (*Euterpe oleracea*, Mart.) were already tested in rodent models of MeHg intoxication showing protection against the deleterious effects of the metal on reproductive system, memory acquisition and visual function, respectively [22,23,24].

Preliminary results with *Euterpe oleracea* support this fruit as an excellent candidate to combat the deleterious consequences of MeHg exposure, due to its remarkable antioxidant properties and the preservation of visual function [24,25]. *E. oleracea* Martius, family Arecaceae, is a common palm in the eastern Amazonian floodplains and its fruits are highly consumed by the Amazonian populations, generally as a thick juice called as the fruit, “açaí”. This juice can be also filtered to eliminate fibers, lipids and proteins (named clarified juice) for commercial use [25]. Clarified juice of *E. oleracea* (EO) is available on the international market and is often used as a base for energy drinks [26]. EO already demonstrated a potent neuroprotective effect with conditions as serious as seizures or depression [25,27]. Although initially attributed to its antioxidant properties, both in vitro and in vivo studies revealed that EO is able to act in diverse targets, such the telomerase reverse transcriptase (TERT) mRNA expression [27]. The TERT is the enzyme responsible for telomere length, presently considered as the biological clock in the countdown of the cells’ senescence. The adequate activity of TERT is essential for maintaining the telomere length, playing a critical role in the control of aging and age-related disorders especially affecting the CNS. In fact, age-related accumulated DNA damage is directly associated with cognitive decline (reviewed by [28]).

Despite the preliminary promising data, it remains unclear whether EO samples for human consumption could show any effect against the MeHg-induced neurotoxicity. Additionally, no data on the possible chelating effect of mercury is available in the literature. Thus, the objective of this work was to verify the effect of EO on the neurobehavioral performance (open field, rotarod and pole tests), the biochemical (lipid peroxidation and nitrite levels in the brain) and aging-related (TERT mRNA expression) parameters, as well as the metal pharmacokinetics (mercury content in the brain) after an acute exposure to MeHg.

## 2. Materials and Methods

### 2.1. Animals

A total of 56 male Swiss mice (20–30 g) were kept under controlled light and temperature (22 ± 2 °C; 12 h light/dark cycle) with food and water ad libitum. Animals were handled properly to reduce stress. The experimental procedures followed the National Institutes of Health (NIH) Guide for the Care and Use of Laboratory Animals and were approved by the Ethics Committee on Experimental Animal Research of the Federal University of Pará (license number BIO 91-15).

### 2.2. Clarified Açaí (Euterpe Oleracea, Mart.) Juice for Human Consumption

Commercial samples of clarified juice of *E. oleracea* (EO) were obtained with a patented process (PI 1003060-3) developed by Amazon Dreams and the Federal University of Pará. Clarification process is very common in food industry, mainly in juice processing. The main concept of clarification is the separation of lipids, proteins, tannins and/or fibers, once they use to flocculate or precipitate during the juice storage. Succinctly, fresh fruits of *E. oleracea* were harvested and cleaned and pulping was performed with the addition of 0.5 L of water per kilogram of fruits. This mixture was subsequently microfiltered (using diatomaceous earth) and centrifuged on a continuous centrifuge to obtain a thin, translucent, wine-colored liquid with no fibers, proteins or lipids (total solids <1% DM) [25].

### 2.3. Analysis of the Juice Composition

The analysis of anthocyanins and major flavonoids content in EO was performed using two validated UHPLC-DAD methods, as described elsewhere [29,30]. Orientin, homoorientin, taxifolin, cyanidin 3-glucoside and cyanidin 3-rutinoside (Extrasynthèse, Genay, France) were used as standard compounds.

### 2.4. Treatments and Samples Collection

**Figure 1:**
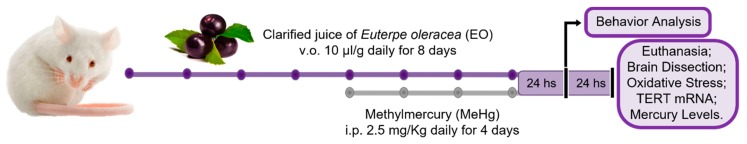
Experimental design. Adult male mice were treated with clarified juice of Euterpe oleracea (EO) or saline by gavage for 8 days. From the fourth day, animals also received intraperitoneally methylmercury (MeHg) or phosphate-buffered saline solution once daily.

The animals were randomly grouped and orally treated once daily with commercial EO or saline (10 μL/g body weight (bw)) for 8 days, by gavage as previously described [25]. From the fourth day (Figure 1), all animals also received intraperitoneally the same volume of phosphate-buffered saline solution (PBS) or MeHg (diluted in PBS, final dose of 2.5 mg/Kg bw) once daily [31]. Twenty-four hours after the last treatment, behavioral analysis were performed and, 24 h later, all animals were euthanized with a mixture of xylazine (5 mg/kg) and ketamine (80 mg/kg) (Figure 1). Brains were immediately dissected and frozen with liquid nitrogen. A set of animals (8–10/group) was evaluated for behavioral analysis and mercury content in brains and a different set of animals (6–8/group) was used for biochemical analysis. For this latter analysis, the brain tissue was homogenized in cold Tris-HCl (1:10 *w*/*v*) and immediately tested.

### 2.5. Open Field Test

The spontaneous locomotor activity was evaluated in an open field arena [32]. The apparatus consists of a square wood, with the floor divided into 25 squares. The mice were placed in the center of the arena for 5 min and the number of vertical exploratory behaviors (rearing) and the total distance performed were recorded using a video monitoring system with ANYmazeTM version 4.99 software (Stoelting Co. , Wood Dale, IL, USA).

### 2.6. Rotarod Test

To evaluate motor coordination, the rotarod test was used [32]. The apparatus (Insight^®^, EFF-411, Ribeirão Preto, Brazil) consists of an acrylic box with a grooved metal roller (8 cm in diameter), with separated compartments (9 cm wide and 16 cm high). During the habituation phase, each mouse was placed on the rotating rod at 8 rpm for 120 s with 300 s rest. Then, three more trials of 120 s with 300 s of rest were performed at 8 (trial 1), 16 (trial 2) and 20 rpm (trial 3). The time for the first drop (latency) in each session was recorded.

### 2.7. Pole Test

The pole test evaluates movement disorders (i.e., bradykinesia) [33]. The animals were positioned on a vertical wooden pole (2 × 50 cm) resting on a circular platform (1 cm; *r* = 25 cm). The time to exit the wooden pole was recorded in five exposures of 120 s with 60 s of rest. For the animals that fell off the platform or did not rotate, the maximum time (120 s) was recorded. Based on the three best times of each mouse, the mean was calculated.

### 2.8. Quantitation of the Total Protein Content

To analyze the total protein content, the Bradford method was used [34], based on the interaction of the Bradford reagent (5% ethanol, 8.5% 176 phosphoric acid, 0.25% Coomassie Brilliant Blue G-250) with the basic or aromatic side chains of the proteins. Samples of brain homogenates were centrifuged at 5600 rpm for 10 min at 4 °C. The supernatants were incubated with Bradford reagent (1:1) for 5 min at room temperature. The absorbance was measured at 590 nm and the results were compared with standard solutions of bovine serum albumin.

### 2.9. Assay of Lipid Peroxidation

The lipid peroxidation of the samples was determined according to Esterbauer and Cheeseman [35], quantifying the chromogenic compound produced by the interaction between malondialdehyde (MDA) and N-methyl-2-phenylindole (NMFI) in the presence of methanesulfonic acid. The samples were centrifuged at 5600 rpm for 10 min. A solution of 325 μL of NMFI (10.3 mM in acetronitrile) diluted in methanol (650 μL) and 75 μL of methanesulfonic acid were added to 100 μL of samples homogenates or standard solutions with MDA and incubated for 40 min at 45 °C. Absorbance was recorded at 570 nm and the data were normalized using the total protein content of each sample. The results were expressed as nmol of MDA per milligram of protein.

### 2.10. Quantitation of Nitrite Levels

The production of nitric oxide was evaluated indirectly by the quantitation of nitrite levels of the samples, as previously described [36]. Samples of homogenates were centrifuged at 14,000 rpm for 10 min. The supernatants were then incubated with sulfanilamide and N-(1-naphthyl) ethylenediamine hydrochloride (1:1) for 20 min at room temperature. Absorbance was measured at 540 nm and compared to standard concentrations of sodium nitrite. The data were normalized using the total protein content of each sample and expressed in μmol of nitrites per mg of protein.

### 2.11. Telomerase Reverse Transcriptase (TERT) mRNA Expression in the Brain

TERT mRNA expression was determined by two-step quantitative reverse transcription PCR (RT-qPCR) using the TaqMan Gene Expression Assay, as previously described [27]. Briefly, total RNA was extracted with the Tri Reagent (Applied Biosystems, USA) following the manufacturer’s instructions. A NanoDrop spectrophotometer (Kisker, Germany) was used to evaluate the RNA concentration and quality along with 1% agarose gels. The synthesis of complementary DNA was performed using the High-Capacity cDNA Reverse Transcription Kit (Applied Biosystems, Poland). RT-qPCR was performed using StepOnePlus equipment (Applied Biosystems, Brazil) with TaqMan^®^ Universal PCR Master Mix and TaqMan probes (Applied Biosystems, Brazil). The GAPDH gene was selected as an internal control for RNA input and reverse transcription efficiency. All qRT-PCR reactions were run in triplicate with a final volume of 10 μL for the target gene (TERT: Hs00972656_m1) and the internal control (GAPDH: NM_002046.3), for 40 cycles, using the standard cycling conditions of the machine. Relative quantification of the gene expression was calculated by the ΔΔCt method and expressed as the fold change proposed elsewhere [37]. In the present study, the control group was designated as the calibrator.

### 2.12. Assay of Mercury Content in the Brain

The quantitation of total mercury (THg) in the samples was performed as proposed elsewhere [38]. In this procedure, about 180 mg of each sample was weighed in 50 mL volumetric flask (PyrexR, Jundiaí, Brazil. For digestion of the samples, two steps were performed: first, a combination of deionized water (Milli-Q Millipore^®^ purification system, Darmstadt, Germany and concentrated acid H_2_O-HNO_3_-HClO_4_-H_2_SO_4_ (1:1:1:5) was added to the samples, then heated in a hot plate at 230 °C for 30 min. The volume was finally filled to 50 mL with deionized water. THg measurement was performed by cold vapor atomic absorption spectrometry (CVAAS) in a Mercury Analyzer Hg-201 (Sanso Seisakusho Co., Tokyo, Japan). Blanks and certified reference material (DOLT-4) were tested to ensure the reliability and reproducibility of results.

### 2.13. Statistical Analysis

For all statistical analysis, *p* < 0.05 was considered significant. The Kolmogorov–Smirnov and Bartlett tests were used to analyze the Gaussian distribution and homoscedasticity of the data, respectively. Parametric and homoscedastic data were expressed as mean ± SEM and analyzed with ANOVA followed by Tukey post hoc test, when appropriated. Non-parametric and/or heteroscedastic data were presented as median and interquartile ranges and tested with Kruskal–Wallis followed by Dunn´s post hoc test, when appropriated.

## 3. Results

### 3.1. Composition of the Euterpe Oleracea Juice for Human Consumption

The content in anthocyanins and the major flavonoids of EO are presented in Table 1. Commercial samples of EO contained 1662.15 mg/L of total polyphenols. The content in anthocyanins was 701 mg/L. The most abundant phenolic compound in the blend was the cyanidin 3-rutinoside, followed by orientin, taxifolin and homoorientin (Table 1).

### 3.2. Behavioral Analysis

**Figure 2:**
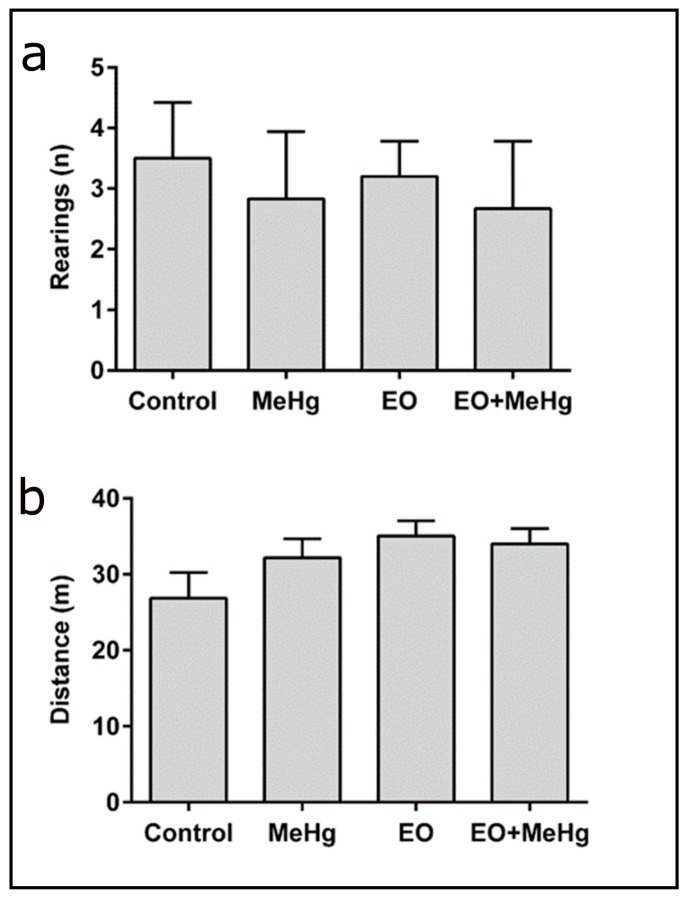
Spontaneous motor performance tested by open field test. No significant difference was observed in the number of rearings (**a**) and the total distance performed (**b**) by mice treated with clarified *Euterpe oleracea* juice (EO) once a day for 8 days and/or methylmercury (MeHg) for 4 days. Data are presented as mean ± SEM (*n* = 8–10).

**Figure 3:**
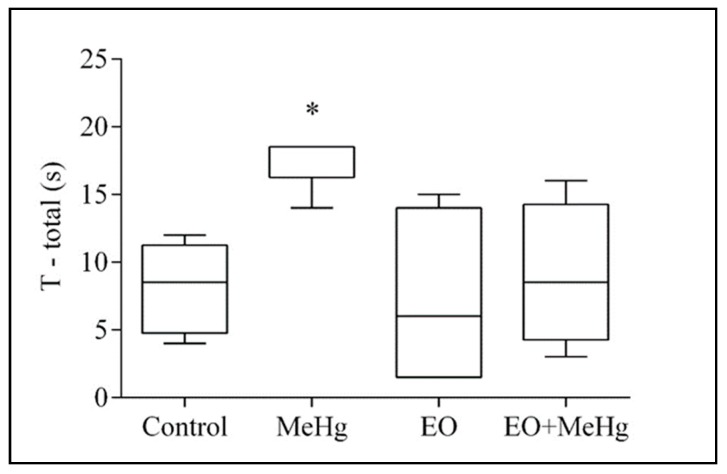
*Euterpe oleracea* consumption totally prevents the alterations caused by methylmercury in the time to descend (latency) in the pole test. Mice were treated with clarified *E. oleracea* juice (EO) once a day for 8 days and/or methylmercury (MeHg) for 4 days. Data are presented as median and interquartiles (*n* = 8–10). * *p* < 0.05 vs all groups.

**Figure 4:**
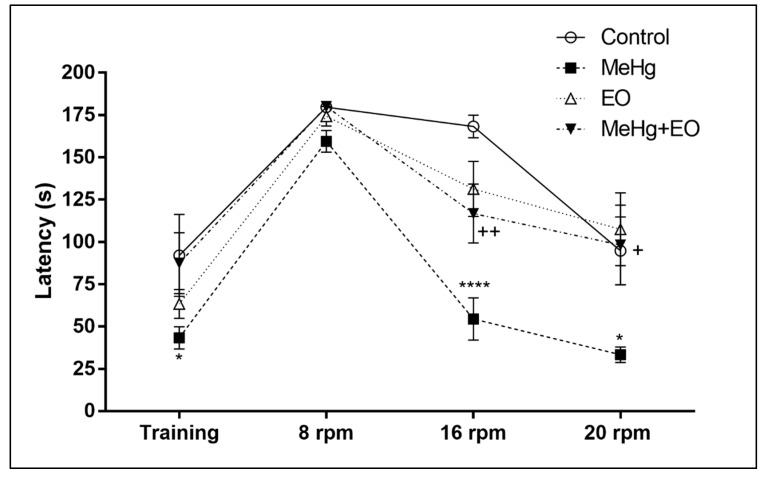
*Euterpe oleracea* consumption significantly reduces the altered motor coordination in rotarod test. Mice treated with clarified *E. oleracea* juice (EO) once a day for 8 days and/or methylmercury (MeHg) for 4 days showed different responses in the latencies to the first fall in the training phase and the trial phases. Data are presented as mean ± SEM (*n* = 8–10). **** *p* < 0.0001 * *p* < 0.05 vs control; ^++^ *p* < 0.01 and ^+^ *p* < 0.05 vs MeHg group.

Behavioral analysis revealed that the consumption of commercial EO does not alter the spontaneous locomotor activity and motor function, as shown by the results of the open field, rotarod and pole tests (Figure 2, Figure 3 and Figure 4). No significant difference was detected in the open field test with the MeHg treatment (Figure 2). However, exposure to MeHg caused a significant increase in time to complete the pole test (Figure 3).

In addition, the MeHg exposure induced poor performance in the rotarod task (Figure 4). In the training phase, it reduced the latency of the first drop when compared to the control animals (Figure 4). This poor performance was also observed when we intensified the task, accelerating the rotation bar (Figure 4).

Interestingly, consumption of commercial EO avoided the motor disturbance caused by MeHg, effectively preventing the difficulty that MeHg-intoxicated animals presented to perform the tasks of the pole and rotarod (Figure 3 and Figure 4).

### 3.3. Analysis of Oxidative Stress

**Figure 5:**
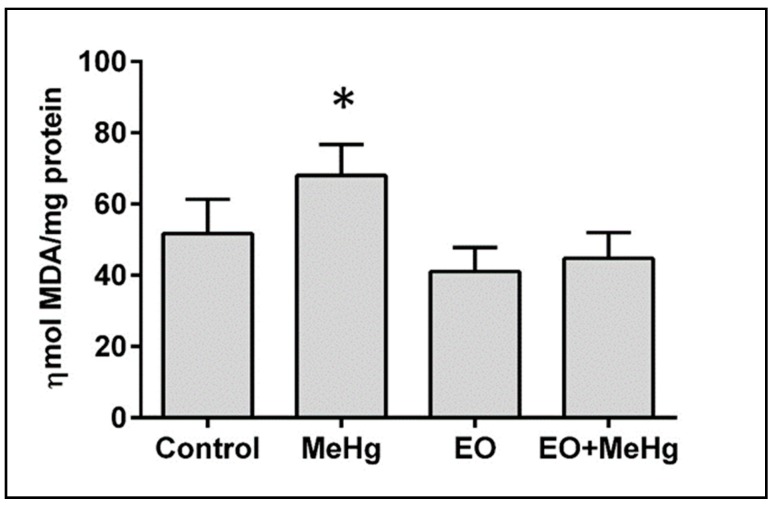
*Euterpe oleracea* consumption prevents lipid peroxidation in brain caused by methylmercury. Mice were treated with clarified *E. oleracea* juice (EO) once a day for 8 days and/or methylmercury (MeHg) for 4 days. Data are presented as mean ± SEM (*n* = 6–8). * *p* < 0.05 vs all groups.

The brains of animals exposed to MeHg showed deleterious consequences of oxidative stress with high levels of lipid peroxidation that were completely eliminated by EO consumption (Figure 5).

**Figure 6:**
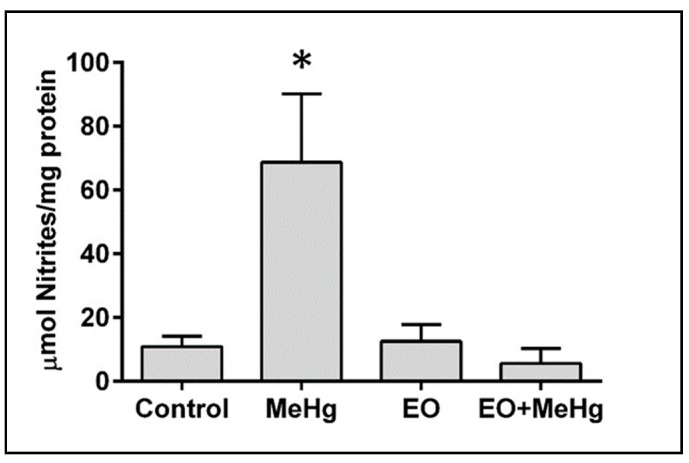
*Euterpe oleracea* protects against methylmercury-induced nitrites production. Mice were treated with clarified *E. oleracea* juice (EO) once a day for 8 days and/or methylmercury (MeHg) for 4 days. Data are presented as mean ± SEM (*n* = 6–8). * *p* < 0.05 vs all groups.

Increase of lipid peroxidation in the brain was associated with increased levels of nitrites (an indirect marker of nitric oxide production) with the MeHg exposure (Figure 6). Treatment with EO showed a potent protective effect and nitrite levels in these animals were similar to those of the control group, pointing to the important role of nitric oxide production in the protection of EO against MeHg.

### 3.4. Telomerase Reverse Transcriptase (TERT) mRNA Expression in the Brain

**Figure 7:**
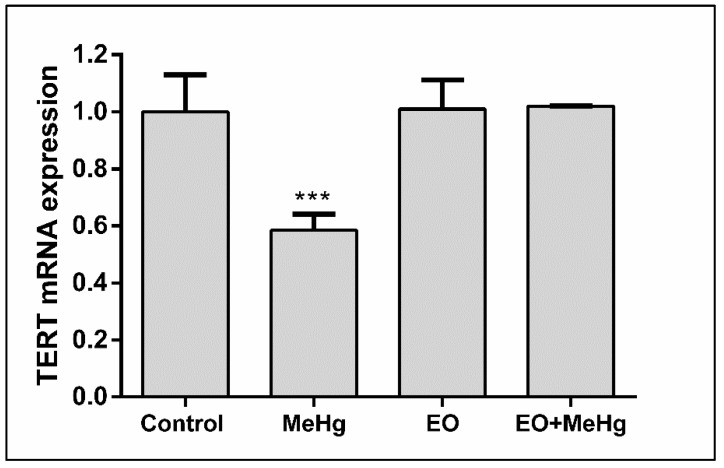
*Euterpe oleracea* juice for human consumption prevents reduced levels of telomerase reverse transcriptase (TERT) mRNA expression caused by methylmercury exposure. Mice were treated with clarified *E. oleracea* juice (EO) once a day for 8 days and/or methylmercury (MeHg) for 4 days. Fold change data are presented as mean ± SEM (*n* = 6–8). *** *p* < 0.001 vs all groups.

TERT expression in the brain was strongly affected by the exposure to the metal, reducing the level of the TERT mRNA to 55.8% (±5.5%) of the levels presented by the brains of non-exposed animals (Figure 7). This significant deleterious effect of mercury was entirely prevented by the consumption of EO.

### 3.5. Analysis of Mercury Content in the Brain

**Figure 8:**
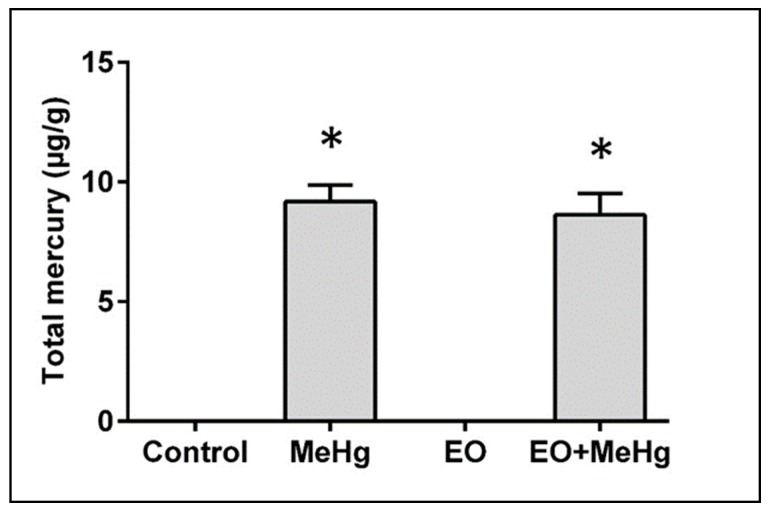
*Euterpe oleracea* consumption does not alter total mercury content in brain. Mice were treated with clarified *E. oleracea* juice (EO) once a day for 8 days and/or methylmercury (MeHg) for 4 days. Data are presented as mean ± SEM (*n* = 6–8). * *p* < 0.05 vs control and EO groups.

To verify if the potent neuroprotective effect of EO was due to possible changes in the mercury toxicokinetics (i.e., distribution and elimination of the metal), Total mercury levels in the brain were assessed (Figure 8). No difference was detected between the mercury content in the exposed animals, treated or not with commercial EO, suggesting that the protective effect of EO against MeHg is not a pharmacokinetic but pharmacodynamic influence (i.e., EO would act to limit the consequences of mercury exposure without influencing the levels of the metal in the CNS).

## 4. Discussion

This work demonstrated that EO juice for human consumption has a potent neuroprotective effect against mercury exposure, with an important role in the protection of motor function and the production of nitric oxide, without apparently altering the toxicokinetics of the metal. Moreover, for the first time, a pronounced deleterious effect of MeHg exposure in the expression of TERT was demonstrated that was entirely prevented by the treatment with EO.

Brain is the main target organ for MeHg. The lowest observed adverse effect level (LOAEL) for the most common neurotoxic effects of MeHg, such as paresthesia, is 50 µg/g of mercury in hair [39]. This level in hair corresponds to approximately 1 µg/g of mercury in the brain, according to the ratio of 250:5:1 for hair:brain:whole blood contents [40]. In our work, the content of mercury in the brains of exposed mice was more than eight times that level, i.e., 8.63 µg/g (Figure 7), characterizing an acute exposure where the deleterious neurological effects of the metal would be totally established. This type of exposure is relevant for humans since it is common to find high levels of exposure in isolated/remote populations such as those of the Amazon [2,5,7,8]. In these populations, as many others around the world, the exposure is mainly through consumption of MeHg-contaminated fish [2,6]. Although partial demethylation of the MeHg can be performed by the gut bacteria, human studies demonstrated that more than 80% of hair mercury is in the form of MeHg [2,5,7,8]. This would be in agreement with in vivo studies confirming that even when animals are orally exposed to MeHg, they show predominant fractions of mercury as MeHg in all tissues [41]. Interestingly, the brain showed the highest percentage as MeHg, being 91.2% as MeHg versus 8.8% as inorganic mercury [41]. In primate models such as squirrel monkeys treated with oral doses of MeHg, the presence of inorganic mercury in the brain was also less than 10% [42]. In vitro studies also confirmed that human cells of CNS origin exposed to MeHg are able of accumulating this metal mainly in the organic form [43].

Considering that we used an animal model exposed to a dose above the LOAEL, it is somewhat surprising that the open field test did not show significant alterations caused by MeHg exposure (Figure 2). However, previous studies also did not observe behavioral changes with this test in animals exposed to similar doses [44,45], supporting that this test may not be sensitive enough to show the many deleterious neurobehavioral consequences. Actually, the performance in the open field test is mainly related to the motor cortex [46]. In previous works with chronic exposure to MeHg, we demonstrated that mercury seems to be less present in the motor cortex and hippocampus in comparison with other areas of the brain, such as prefrontal cortex and cerebellum, of exposed animals [47,48]. In this sense, our hypothesis is that even with higher doses of MeHg, the four-days exposure in adult male mice used in this work is not sufficient to affect the motor cortex to produce significant changes in spontaneous exploration.

Interestingly, the pole test and the rotarod test detected significant changes with the MeHg exposure (Figure 3 and Figure 4) when the open field test failed, supporting previous data [44] demonstrating that pole and rotarod tests would be better at detecting motor neurobehavioral changes caused by this metal. The pole test is a useful tool to predict bradykinesia, i.e., the presence of slowness of movements and the impaired ability to adjust the body´s position [49]. This phenomenon is closely associated to the striatum and the status of its dopaminergic synapses, being frequently detected in Parkinson´s disease [49]. The rotarod test is classically used to identify motor coordination disorders [50]. This test specifically evaluates the balance, physical condition, coordination and motor-planning, taking advantages from the possibility of increasing the difficulty of the task by accelerating the rotations [50]. Considering that both the pole test and the rotarod would be useful tools to predict the damage of striatal function and dopaminergic cells [49,50,51,52], the above-mentioned facts suggest that mercury exposure may affect dopaminergic neurons (especially in the striatum), supporting the relationship between brain mercury content and Parkinson-like characteristics as previously described in humans and animal models [53,54,55]. Moreover, the rotarod test is also especially sensitive for detecting cerebellar dysfunction [56] and the cerebellum is classically known as one of the primary brain areas affected by MeHg [39]. Our results suggest that behavioral changes related to bradykinesia and motor coordination may occur as hallmarks of intoxication. This would be in agreement with the changes observed in human populations, since the intake of this metal has the potential to cause neurobehavioral abnormalities, especially in psychomotor coordination [14,15,16].

The MeHg-induced neurotoxicity is closely linked to oxidative stress and its subcellular consequences such as lipid peroxidation or genotoxicity [17,18,23,57,58]. A significant increase in lipid peroxidation was detected in the brains of exposed animals, including when no spontaneous neurobehavioral abnormalities were shown in the open field test (Figure 2 and Figure 5). The CNS is especially susceptible to lipid peroxidation due to its high content of polyunsaturated fatty acids. Due to a self-propagating chain reaction, cell membrane lipids are damaged by free radicals, leading to membrane rupture and cell death [18]. Lipid peroxidation responsible for MeHg-induced neurotoxicity may also be associated with increased nitrite levels (as an indirect marker of nitric oxide production) [18,59,60]. It has already been demonstrated that some of the initial effects of MeHg on cells of CNS origin occur via activation of the nitrergic system [60,61,62]. Here, we found increased nitrite levels in the brains of the exposed animals (Figure 6), confirming this activation of the nitrergic system as a relevant effect of MeHg-induced neurotoxicity (when no major change was detected in spontaneous neurological behavior).

One of the main targets of oxidative stress caused by mercury is DNA. Additionally, this metal is able of affecting genomic instability by other mechanisms such as microtubule disruption and direct interaction [17]. The damage to this macromolecule has been well-stablished, even with exposure to very low concentrations of the metal, as revealed by pre-clinical and human studies [17,57,63,64]. MeHg exposure causes high frequencies of genomic instability biomarkers, including micronuclei (which are biomarkers of whole chromosome loss and breakage), being cells of CNS origin especially susceptible to this damage [57,64]. In this scenario, the adequate activity of the DNA maintenance and repair systems plays an essential role to minimize the long-term consequences of the exposure to this metal. Human studies already demonstrated an impartment DNA repair ability in adults and children exposed to mercury forms [65,66]. However, results about the influence of this metal in the expression of enzymes such as 8-oxoguanine DNA glycosylase are conflicting [66,67,68], perhaps due to confounding factors such as age or exposure to multiple pollutants. Therefore, more pre-clinical studies with controlled conditions are necessary to better understand the possible deleterious influence of the metal in the adequate DNA preservation.

In this scenario, the maintenance of telomeres length is essential for the genomic stability, protecting the end of chromosomes and preventing the premature aging syndrome. This function is mainly carried out by the TERT. Literature about the possible influence of the metal is extremely scarce. Only two recent studies with exposed human populations demonstrated contradictory results in the analysis of telomere length in blood [69,70]. However, blood is not the main target organ for MeHg and confounding factors in these studies are still an open question. To our knowledge, this is the first time that a pre-clinical study on mercury toxicity is carried out to evaluate this question. As possible differences in telomere length could not be detected because of the short time of exposure, we focused in the TERT mRNA expression, a sensitive parameter that better reflects the long-term consequences of the exposure. Remarkably, MeHg exposure reduces the expression about a half, characterizing a status susceptible to premature aging syndrome with cognitive impairment and oncogenic processes [28,71].

It is relatively common for antioxidant compounds to be tested as possible protective therapies against exposure to MeHg [18]. Although many of these compounds have already demonstrated a potent effect, the access of isolated/remote populations (such as riverine inhabitants of the Amazon) to isolated molecules is very difficult. This is due to the characteristics of these populations (precarious health care system, low purchasing power, geographical isolation, etc.) [2,5,7,8,63,72], similar to those of other vulnerable populations in the world. Thus, the use of endemic plants is a more realistic alternative for these populations to protect themselves against MeHg-induced neurotoxicity. In recent years, many efforts have been devoted to the analysis of plants used by vulnerable populations in developing countries, such as India or Brazil [20,73,74,75,76,77]. Thus, this work contributes to the need to find alternatives for vulnerable populations exposed to MeHg.

In this sense, preliminary results have shown that *E. oleracea* may be a good candidate to combat MeHg neurotoxicity, both by improving visual function parameters and by potent antioxidant properties, superior to those of ascorbic acid [24,25]. Unfortunately, in the latter works it is unknown what are the main components of EO (important information, because they depend on the soil and the way the açaí pulp is prepared, among other factors) [78]. Thus, the present work tested for the first time commercial samples of EO (of known composition) that guarantee the reproducibility of the preparation and the indicative for human consumption, increasing the relevance and the impact of the results.

In addition, this work avoided common confounding factors, such as the possible direct interaction between the EO components and MeHg using a different exposure pathway for each treatment (gavage and i.p., respectively). MeHg has high affinity for nucleophilic groups (mainly thiols and selenols) that can be found in proteins and in many natural products. The administration of EO and MeHg by the same route could allow a direct interaction avoiding the absorption of MeHg (and therefore the installation of the model). Additionally, EO by gavage, not by free access to food, ensures that all animals receive the same treatment. The EO dose used in this work reproduces the daily intake of human populations in the northern region of Brazil [26].

The neurofunctional protection exerted by the plants is usually associated with the decrease of oxidative stress [73,76,79,80]. Interestingly, the protective effect of EO, completely eliminated all the neurobehavioral, biochemical and age-related changes caused by this acute exposure to MeHg, normalizing motor coordination, lipid peroxidation levels, nitric oxide production and the levels of TERT mRNA expression in the brain (Figure 2, Figure 3, Figure 4, Figure 5, Figure 6 and Figure 7). In fact, the neuroprotection of EO is so potent that, even against worse conditions (such as seizures), it effectively protects the brain [25].

However, the question remains whether this protective effect would be due to changes in clearance/absortion of MeHg (i.e., a pharmacokinetic effect of EO) or due to the influence of EO on the molecular mechanisms of MeHg (i.e., a pharmacodynamic effect of EO). As discussed elsewhere [18], in the search for therapies against MeHg intoxication, it is often unknown whether the protection is mediated by the restoration or prevention of the functionality affected by MeHg or by the decrease in the body burden of mercury. Moreover, lower content in mercury has been associated with high fruits intake in humans, pointing to possible mercury chelating properties that may facilitate metal removal [19]. Thus, the analysis of the mercury content in the brain may be essential in studies evaluating the protective effects to answer the above question. Unfortunately, the inclusion of this analysis is very unusual in the literature on ethnopharmacology for MeHg neurotoxicity. This work demonstrated for the first time that EO does not significantly influence the mercury content in the brain (Figure 8), supporting the idea that the potent protective role of EO would be primarily pharmacodynamic rather than pharmacokinetic. Our work agrees with recent data demonstrating that the in situ antioxidant profile, different in each brain area, plays an essential role in the neurotoxicity of MeHg [81] and that this would predict the deleterious consequences of MeHg exposure for each area better than the exact levels of mercury in the region, since sometimes no correlation between mercury content and neuropathological changes is found [12,81]. Therefore, the control of the oxidative process may be the key against the deleterious changes caused by this metal and reinforcing the antioxidant profile with treatments such as EO juice may be an adequate strategy.

The direct scavenger effect of EO against nitric oxide and other free radicals [25] would help prevent neurotoxicity. Although nitric oxide is not the only free radical that mediates MeHg neurotoxicity, it may play a main role in the protection by EO since the increase in nitrite levels was completely avoided (Figure 6). Although the exact molecular mechanism of EO neuroprotection remains unknown, our data on the main components of EO (Table 1) and oxidative stress (Figure 5 and Figure 6), already point to a possible hypothesis, the influence on the NF-κB pathway. For example, orientin is able to prevent increased nitric oxide production by inhibiting of NF-κB pathway, especially in microglia [82,83]. A similar effect (inhibition of nitric oxide production by blockade of the NF-κB pathway) has also been demonstrated for taxifolin [84]. Moreover, it has been shown that the anthocyanin-rich fraction of *E. oleracea* fruits protects microglial cells against lipopolysaccharide insult by inhibiting cytokine release, nitric oxide production and phosphorylation of NF-κB [85]. Decreased phosphorylation of NF-κB would prevent its translocation to the nucleus and expression of pro-inflammatory mediators such as cyclooxygenase-2 and nitric oxide synthase (the enzyme responsible for the production of nitric oxide). However, this pathway may not explain the increase of TERT mRNA expression caused by EO against mercury exposure. Considering that there is a NF-κB-binding site in the TERT promoter that induces the expression of the enzyme, the NF-κB decrease in the nucleus may be an opposite effect for that. Agreeing with the hypothesis that a different mechanism would be responsible for the effect on the TERT, the magnitude of the effect on the TERT expression seems to be more pronounced (almost 50%) than the oxidative stress caused by the metal. Therefore, additional synergic actions through other pathways cannot be discard. For example, it has recently been demonstrated that this juice would enhance inhibitory transmission by direct interaction with GABAergic targets [86]. The improvement of GABAergic transmission would have a protective effect against oxidative stress, as already demonstrated in other models [87]. Independent of the exact mechanisms, the synergic actions of all components of EO (mainly polyphenols and anthocyanins) may lead to the decrease of free radical nitric oxide (and its consequence, lipid peroxidation) as well as the preservation of the TERT mRNA expression, being responsible for the potent neuroprotective and anti-aging effects observed in our work.

## 5. Conclusions

The acute exposure to MeHg used here was above the maximum levels already detected in the Amazonian populations [2], with the deleterious neurological effects of the metal being totally established. Even under these conditions, consumption of EO juice (similar to the regular intake of human populations) was sufficient to significantly prevent the neurotoxicity induced by MeHg (neurobehavioral, biochemical and aging-related changes). Even with the pronounced decrease detected in TERT expression that was caused by MeHg exposure, EO completely prevented this aging effect, maintaining the levels of the TERT mRNA similar to those of the control group. As revealed by the mercury content in the brain, this was not a pharmacokinetic effect, but essentially a pharmacodynamic effect of the EO juice. This finding would not support previous hypotheses about the possible chelating properties of fruit consumption in the Amazon [19]. This pharmacodynamic effect of the juice was associated with the prevention of oxidative stress with a major role in inhibiting the nitrergic system in the brain. Moreover, EO totally avoided the possible premature aging syndrome caused by mercury exposure, preserving the TERT expression. Our data already support that regular consumption of *E. oleracea* juice may be an excellent option for exposed Amazonian populations to have additional protection against MeHg intoxication.

## Figures and Tables

**Table 1:** Major phenolic compounds of the *Euterpe* *oleracea* juice.

Phenolic Compound	Content (mg/L)	Content (%)
Cyanidin 3-rutinoside	448	63.9% *
Cyanidin 3-glucoside	184	26.2% *
Taxifolin deoxyhexose	308	18.5% **
Orientin	381	22.9% **
Homoorientin	247	14.8% **

* of the total anthocyanins; ** of the total polyphenols.
